# A Systematic Review and Meta-Analysis to Find Out the Efficacy of Socket Preservation Techniques in Adults in Split-Mouth Randomized Controlled Trials

**DOI:** 10.7759/cureus.79873

**Published:** 2025-03-01

**Authors:** Kiruthiga V Pushpanathan, Sabarigirinathan Chitrpautirapillay, Rupkumar Pushparaj, Prasana Kumar

**Affiliations:** 1 Prosthodontics, Asan Memorial Dental College and Hospital, Chengalpattu, IND; 2 Prosthodontics, Government Dental College and Hospital, Chennai, IND; 3 Oral Medicine and Radiology, Adhiparasakthi Dental College and Hospital, Chennai, IND

**Keywords:** alveolar bone loss, alveolar process, alveolar ridge preservation, dental implants, split-mouth design

## Abstract

Alveolar ridge preservation has been practiced for a long time, yet it is not a standard component of post-extraction care. Post-extraction bone loss is an inevitable consequence, but successful implant treatment planning requires adequate bone and soft tissue coverage. Delayed implant placement and fixed partial dentures may significantly benefit from alveolar ridge preservation. This systematic review aimed to gather evidence on alveolar ridge preservation using split-mouth randomized controlled trials. The study protocol was registered in the International Prospective Register of Systematic Reviews (PROSPERO) with the registration ID number CRD42020177085. A comprehensive literature search was conducted using electronic databases and manual searches, yielding 4,654 results, from which 10 eligible studies were selected. These studies included a total of 101 participants and 202 extraction sockets. Meta-analysis was conducted using the fixed and random effects generic inverse variance method with the RevMan 5.3 software (Cochrane Collaboration, London, UK). The analysis revealed that the mean bone dimensional change in width at three months was 1.99 (0.63, 3.35), while the vertical height changes for the buccal and lingual plates were 1.13 (0.57, 1.70) and 0.46 (-0.06, 0.98), respectively. The pooled mean for differences in width at six months favored alveolar ridge preservation, though the internal vertical height changes at six months provided contradictory results. The findings indicate that socket preservation techniques help reduce alveolar bone loss. However, the conclusions must be interpreted cautiously, as further research with long-term randomized controlled trials is necessary to evaluate outcomes beyond bone loss.

## Introduction and background

Following a tooth extraction, a cascade of events ensues. Soon after extraction, a blood clot fills the socket. Thereafter, early, intermediate, and late phase changes occur as the bone remodels. During the first week, granulation tissue formation occurs as the clot shrinks. At three weeks, the woven bone begins to appear. Four to six weeks post-extraction, there is the formation of lamellar bone. Mature bone formation along with corticalization of the entrance of the extraction socket takes place between the fourth and the sixth month. Resorption takes place simultaneously as early as the second phase and continues as the cortical lining of the socket is completely lost [1,2]. Bone resorption never ceases, and it takes place at a slower rate throughout life; the same is dependent on several factors [3,4,5]. However, implant treatment planning mandates adequate bone and healthy periodontium. Not only do these factors play a significant role in aesthetics and function, but also the clinical longevity and success of the implant restoration. There are several methods for managing less-than-ideal bone scenarios. These procedures are used either singly or in combination with others. There is no consensus as to a standard protocol for the prevention of hard and soft tissue deficiency for delayed implantation. Currently, it depends on the treating dentist, individual clinical picture, and patient factors [6,7,8]. The same factors also apply to fixed partial dentures. Ridge augmentation surgery, though effective, carries along with it the additional cost and a further delay before implant placement. The reviews conducted under this topic have concentrated on different outcomes in totality or part. However, there exists extensive clinical and statistical heterogeneity. To minimize some of the statistical heterogeneity, we have undertaken this review with split-mouth randomized controlled trials aiming for totality to clarify this lingering uncertainty that surrounds the role of alveolar ridge preservation (ARP) in restorative rehabilitation.

## Review

Methodology

Study Protocol

This review is in accordance with the Preferred Reporting Items for Systematic Reviews and Meta-Analyses (PRISMA) guidelines [9]. A standard electronic search about this study was performed in PubMed, ProQuest, Cochrane Central Register of Controlled Trials (CENTRAL), WorldWideScience, and Clinical Trials Registry. The search strategy was as follows: (dental implant placement) OR (autograft) OR (allograft) OR (alloplast) OR (plasma-rich fibrin) OR (alveolar ridge preservation) OR (socket preservation) AND (randomized controlled trials) for PubMed. Similar terms with minor modifications unique to each search engine were adopted. An additional manual search was conducted to include possible left-out grey literature and theses, along with a thorough screening of individual journals by cited reference searching. All the above searches were re-run before conducting the final analyses to allow for new additions. The last search was conducted on 14/8/2020. The search filter was not customized for year settings.

Focus question: What is the effect of socket preservation procedures in the adult population on alveolar bone resorption following extraction in comparison to spontaneous healing when the test and control are within the same individual, such as in split-mouth randomized controlled clinical trials?

Eligibility Criteria

The study eligibility criteria included systematically healthy adult participants of both sexes classified as American Society of Anesthesiologists (ASA) class I. Only randomized controlled trials with a split-mouth study design were considered. Participants should not have been under medications affecting bone metabolism. The studies needed to include any grafting intervention for ridge preservation, with the control group adhering strictly to normal healing. The interventions consisted of atraumatic extractions followed by alveolar ridge preservation procedures, such as socket grafting, socket sealing, guided bone regeneration, and growth factors, either used individually or in combination, with treatment planned for delayed implant placement. Comparisons were made with extraction sockets left to heal without any ridge preservation procedures. Studies assessing changes in alveolar ridge dimensions, both horizontal and vertical, as the primary outcome and changes in histology and bone density as secondary outcomes were included.

Exclusion criteria encompassed studies evaluating ridge preservation procedures in impacted third molar extractions, those with evaluations conducted for less than two months, and studies involving traumatic extractions. Articles written in languages other than English were excluded, as were cohort, case-control, and retrospective studies.

Study Selection

**Figure 1:**
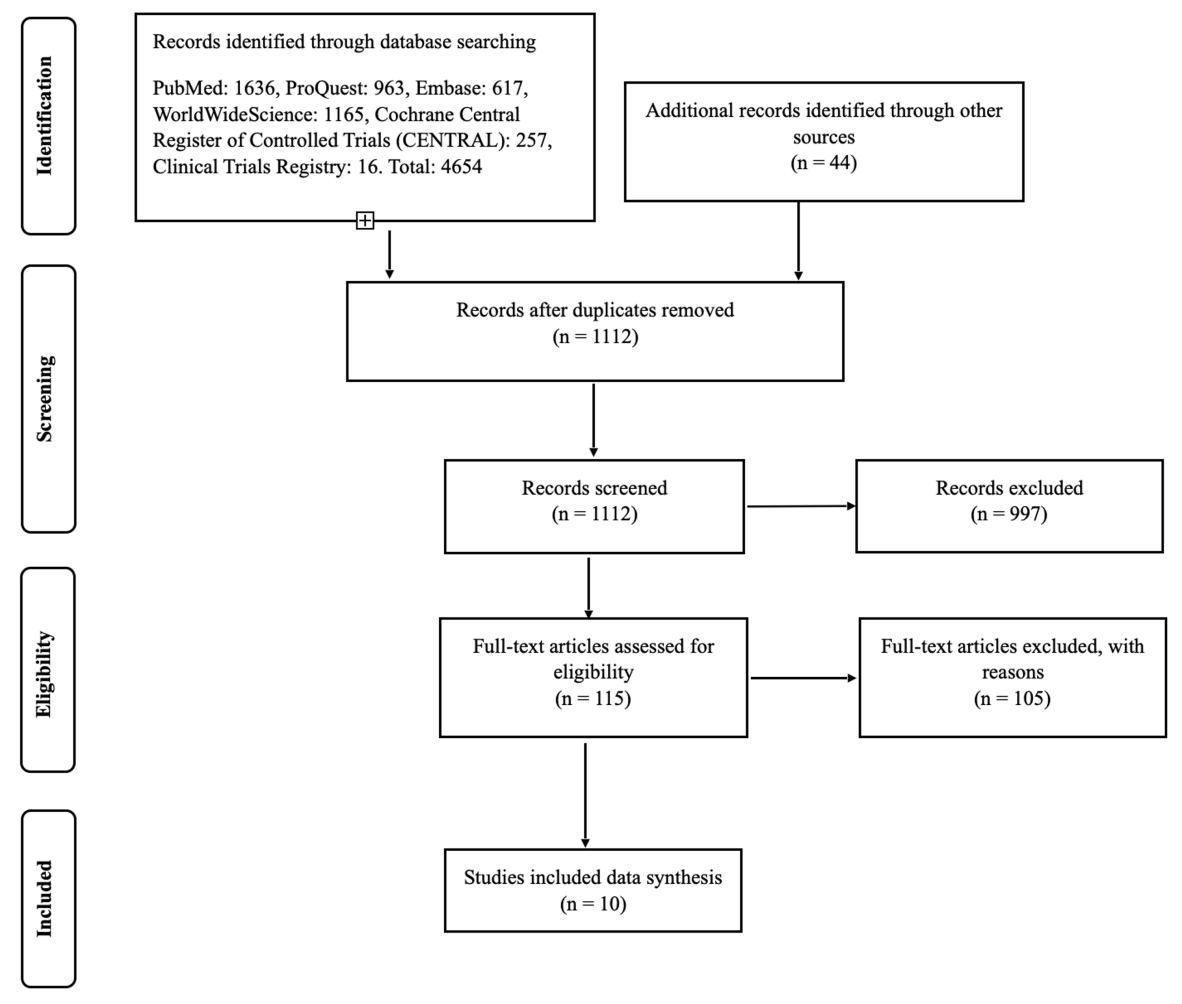
Preferred Reporting Items for Systematic Review and Meta-Analyses (PRISMA) flow diagram depicting selection process Embase: Excerpta Medica Database

A total of 4,654 articles were obtained from the above-mentioned sources. A PRISMA flow diagram depicts the selection process (Figure 1).

After the omission of duplicate records, a total of 1,112 articles were available for the selection process. Two reviewers (KVP and SN) individually assessed for possible eligibility following a pre-drafted checklist in a sequential manner from title selection to abstract selection and, finally, a full-text evaluation. They were not blinded to the names of the journals and authors. An article was chosen in the case of agreement between the two. Any differences in opinion about the inclusion of an article were resolved by discussion. The kappa statistic for the inter-rater agreement was 0.70. The whole exercise was repeated once to eliminate errors and biases before finalizing the selection list. The reason for exclusion was noted and saved for future reference. The excluded studies, along with reasons for exclusion, are depicted in Table 1.

**Table 1:** Studies excluded from the review

Sl. No	Study ID	Reasons for exclusion
1.	Nunes et al. (2018) [10]	Membrane was used in the control group
2.	Pinho et al. (2006) [11]	Membrane was used in the control group
3.	El Shazley et al. (2016) [12]	Adolescent population
4.	Joshi et al. (2017) [13]	Membrane was used in the control group
5.	Srinivas et al. (2018) [14]	Controlled clinical trial without randomization
6.	Lekovic et al. (1997) [15]	Case report series
7.	Camargo et al. (2000) [16]	Controlled clinical trial without randomization
8.	Stoppenbrink et al. (2019) [17]	Adolescent population
9.	Kerr et al. (2008) [18]	Ultrasound was used for alveolar ridge preservation, a preliminary study
10.	Daugela et al. (2018) [19]	A study was done on impacted 3^rd ^molar teeth, and the follow-up does not meet the eligibility criteria
11.	Crespi et al. (2016) [20]	Reactive soft tissue in the preservation of large bone defects
12.	Pelegrini et al. (2010) [21]	Bilateral extractions but tests and controls allocated in different patients
13.	Reichert et al. (2014) [22]	The follow-up was six weeks, and the age group of the population
14.	Kollati et al. (2019) [23]	The ages of the included patients ranged from 17 to 50 years

Data Extraction

Two authors participated in data extraction. The included studies were subject to data collection. A detailed table with the following headings was prepared according to the guidelines outlined by the Cochrane Collaboration for data collection [24]. The headers were as follows: identity: the author, and year of publication; study characteristics: IRB registration, study setting, country/region, study design, study arms, inclusion, and exclusion criteria, sampling procedures, confounders, randomization, allocation concealment, blinding parameters, and follow-up evaluation; intervention: methods of tooth extraction, grafting procedure, graft material, flap, healing (primary/secondary), antibiotic coverage, maintenance therapy, teeth involved/socket morphology, clinical analysis-evaluation tools, method of evaluation, unit of evaluation, and adverse effects; statistical characteristics: sample calculation, method, outcome measure, significance, and dropouts.

All the published articles, along with the figures and graphs, were collected for data retrieval. Additional information was sought from trial registries and the entire thesis when and where possible.

Quality Assessment

The RoB Version 2 tool of Cochrane Collaboration (London, UK) was utilized to identify and classify studies according to the risk of bias [25]. This tool is an improvisation of RoB 1, which was designed exclusively for risk assessment in randomized controlled trials. This newer version has a series of signaling questions with response options and the risk of bias judgments. Two authors (RK and KVP) assessed the risk of bias. Each study was subjected to assessment for all domains and categorized as low, unclear, and high risk accordingly. Any disagreements were resolved through discussion, and consensus was achieved. Unresolved conflicts were arbitrated by the third author (PK).

Meta-Analysis

Studies were analyzed for clinical heterogeneity, and only those that were homogenous were pooled together. Only the primary outcome was homogenous enough to be subjected to quantitative analysis. Both fixed and random effects models were utilized depending on the amount of statistical heterogeneity. The chi-squared and I² tests were employed to test for heterogeneity [26]. A random effects model was chosen if the I² values were between 50% and 100%. A generic inverse variance analysis was performed to estimate the overall effect sizes. The standardized mean difference was employed only when different studies employed different scales.

Results

Quantitative Analysis

Study selection: A total of 4,654 articles were obtained as the combined effort of the searches. After the sequential selection process, only 10 articles were deemed fit to be considered as included articles [27-36]. Of these, only five articles were eligible for meta-analysis. They are as follows: Temmerman et al. (2016) [27]; Jung [28]; Madan [30]; Festa [34]; and Lekovic [36]. All these studies yielded a total of 101 study subjects (202 extraction sockets).

Study characteristics: All the studies were well within the inclusion and exclusion criteria. The grouped studies were randomized controlled trials. All were two-arm studies. Two of the studies had symmetric bilateral extractions [28,34], whereas the other studies had a close-to-symmetric extraction [27-30]. They were conducted on people above the age of 18 years. The change scores were obtained at six months for all the pooled studies, except one study that evaluated results in the third month [27]. In another study, the differences were analyzed in the third and sixth months [28]. The control groups in all the included trials were allowed to heal without any intervention. Alveolar ridge preservation by socket grafting was employed in four of the included studies, whereas, in one study, a growth factor, leukocyte-platelet-rich fibrin clots, along with socket sealing by advanced platelet-rich fibrin, was utilized [27]. The graft material varied between the studies. Two studies used a xenograft and a membrane for socket preservation [28,31]. A membrane alone was employed in one study [36] and an alloplast in one other study [30]. All the pooled studies assessed dimensional changes. In three of the studies, cone-beam computed tomography (CBCT) was the assessment tool [27,28,30]. The other two studies employed direct measurements using a probe soon after extraction and during re-entry surgery [34,36]. Descriptive statistics are presented as mean and standard deviation. A concise table of study characteristics with important aspects is presented for reference in Table 2.

**Table 2:** Characteristics of included trials

S. no	Study name	Age	Intervention	Comparison	Evaluation	Outcome	Sample size	Excluded patients	Follow-up	Teeth involved
1	Temmerman et al. (2016) [27]	54 ± 11 (Mean ± SD)	Intervention: Platelet-rich fibrin (PRF) clot leucocyte and platelet-rich fibrin (L-PRF) membrane; Suture: Crossed horizontal mattress	Control: Spontaneous healing; Suture: Crossed horizontal mattress	A. Radiographic analysis, cone beam computed tomography (CBCT). B. Pain assessment: McGill pain questionnaire	Vertical dimension, horizontal dimension, socket fill pain	22 (15 Males, 15 Females)	None	3 months	Uni-radicular teeth (premolar, canine, incisor, maxillary and mandibular teeth)
2	Jung et al. (2018) [28]	>18 Years	Intervention: Deproteinized bovine bone mineral + 10% collagen (DBBM-C), native bilayer collagen membrane (NBCM); Suture: Crossed sutures	Control: Spontaneous healing; Suture: No	A. Radiographic analysis, cone beam computed tomography (CBCT)	Vertical dimension, horizontal dimension	18	4	3 months and 6 months	Premolar molar (maxillary and mandibular teeth)
3	Suttapreyasri et al. (2013) [29]	≥ 20 Years - 22.62 ± 2.44 (Mean ± SD)	Intervention: Platelet-rich fibrin (PRF) clot; Suture: Figure of 8	Control: Spontaneous healing; Suture: Figure of 8	A. Clinical direct measurement-study cast + acrylic jig + measuring microscope. B: Radiographic analysis–intraoral periapical radiograph (IOPA)	Vertical dimension, horizontal dimension, soft tissue healing	8 (5 Males, 3 Females)	None	2, 4, 6, and 8 weeks	Premolars (maxillary and mandibular teeth)
4.	Madan et al. (2014) [30]	20 to 45 years	Intervention: Polylactide-polyglycolide sponge, synthetic resorbable alloplastic material; Suture: Crossed horizontal mattress	Control: Spontaneous healing; Suture: Crossed horizontal mattress	A. Radiographic analysis, cone beam computed tomography (CBCT). B. Histology	Vertical dimension, horizontal dimension, bone density, histology	15 (7 Males, 8 Females)	None	6 months	Central and lateral incision canine premolars
5	Crespi et al. (2009) [31]	28 to 72 years, Mean: 51.3 years	Intervention: Magnesium enriched hydroxyl-apatite (MHA) collagen membrane; Suture: Yes	Control: Spontaneous healing; Suture: No	A. Radiographic analysis–intraoral periapical radiograph (IOPA). B. Histology	Vertical dimension, histology	15 (8 Males, 7 Females); 45 sockets (A-15, B-15, C-15)	None	3 months	Molars and premolars (maxillary and mandibular teeth)
6	Karaca et al. (2015) [32]	36 to 60 years, Mean: 46.7 years	Intervention: Free gingival graft; Suture: Yes	Control: Spontaneous healing; Suture: No	A. Radiographic analysis, cone beam computed tomography (CBCT). B. Clinical evaluation–soft tissue healing	Vertical dimension, horizontal dimension	10 (5 Males, 5 Females)	None	3 months	Incisors and canine (maxillary teeth)
7	Araujo-Pires et al. (2016) [33]	≥ 18 Years	Intervention: Poly (DL-lactide-co-glycolide/calcium phosphate) small and large titanium screws; Suture: Vertical mattress	Control: Spontaneous healing; Suture: No	A. Radiographic analysis, cone beam computed tomography (CBCT), and micro-CT. B. Histology	Vertical dimension, horizontal dimension, bone volume, trabecular number, thickness and separation, histology	10	None	4 months	Anterior maxillary teeth
8	Festa et al. (2011) [34]	28 to 58 years	Intervention: Cortico-cancellous porcine xenograft, soft cortical membrane; Suture: Yes	Control: Spontaneous healing; Suture: No	A. Clinical measurement, re-entry surgery-soft tissue parameters (adjacent teeth)	Vertical dimension, horizontal dimension, bleeding on probing pocket depth recession	15 (6 Males, 9 Females)	None	6 months	Premolars (maxillary and mandibular teeth)
9	Alsayed et al. (2020) [35]	≥ 20 years	Intervention: Platelet-rich fibrin (PRF) clot; Suture: Mattress suture	Control: Spontaneous healing; Suture: Mattress suture	A. Clinical measurements –probe radiographic analysis, cone beam computed tomography (CBCT)	Vertical dimension, horizontal dimension bone density	20 (11 Males, 9 Females)	None	6 months	Premolars
10	Lekovic et al. (1998) [36]	52.6 ± 11.8 (Mean ± SD)	Intervention: Bio-absorbable membrane (glycolide and lactide polymer); Suture: Vertical mattress and interrupted sutures (horizontal incisions)	Control: Spontaneous healing; No membrane	A. Clinical measurement: Initial surgery-re-entry surgery-titanium pins for reference	Vertical dimension, horizontal dimension	16 (10 Males, 6 Females)	None	6 months	Uni-radicular anterior and premolars

Data synthesis: Data for continuous outcomes such as the mean, standard deviation, and the number of participants were collected. Since this review evaluates bone remodeling, the change from the baseline scores was recorded. The reduction in bone resorption was considered a negative means, and the socket fill value was considered a positive means. When the average and variations were not presented in the desired format, missing data were calculated from the published data when and where possible. In the study conducted by Jung [28], change scores were reported for two subgroups, one in the maxilla and one in the mandible, which was combined as a single intervention group. To eliminate the unit-of-analysis error, the recommendation given by the Cochrane textbook of systematic reviews was followed [26]. Only the absolute values were taken into account; the related or adjusted values were not used for analysis. The meta-analysis was carried out with the RevMan 5.3 software of Cochrane Collaborations.

Adverse effects: No adverse effects were reported in the studies conducted by Jung et al. (2018) [28]; Suttapreyasri et al. (2013) [29]; Madan et al. (2014) [30]; Crespi et al. (2009) [31]; Karaca et al. (2015) [32]; Araujo Pires et al. (2015) [33]; Alsayed et al. (2020) [35]; and Lekovic et al. (1998) [36]. Festa et al. (2011) [34], in their study, mentioned mild post-surgical pain and swelling without information on the numbers. Temmerman et al. (2016) [27] reported pain and malodor in two patients of the control group [30]. All of the above-mentioned were treatable, and the patients continued through the entire study.

**Figure 2:**
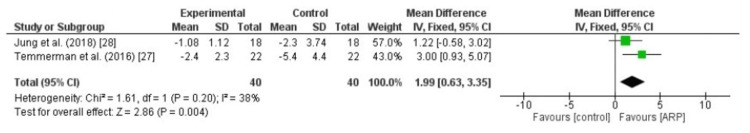
Forest plot depicting pooled effect estimates of horizontal alveolar ridge dimension measured at three months Jung et al. (2018) [28]; Temmerman et al. (2016) [27]

Results of individual studies: Horizontal dimensional changes were assessed in two studies with an outcome of three months. Two studies were pooled (Temmerman et al. (2016) [27] and Jung et al. (2018) [28]) to assess the horizontal width changes at three months (Figure 2). A total of 40 patients with bilateral extractions, accounting for 80 extraction sockets, were studied. The effect size with the 95% confidence interval at three months was 1.99 (0.63, 3.35).

**Figure 3:**
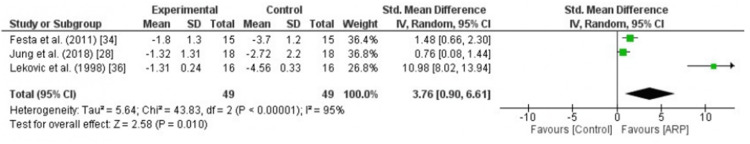
Forest plot depicting pooled effect estimates of horizontal ridge dimensions measured at six months Jung et al. (2018) [28]; Festa et al. (2011) [34]; Lekovic et al. (1998) [36]

Outcome (six months): Three studies were pooled (Jung et al. (2018) [28], Festa et al. (2011) [34], and Lekovic et al. (1998) [36]) to estimate the reduction in the alveolar ridge width (Figure 3). The studies included 49 patients with 98 extraction sockets. The effect size with the 95% confidence interval at six months was 3.76 (0.90, 6.61).

**Figure 4:**
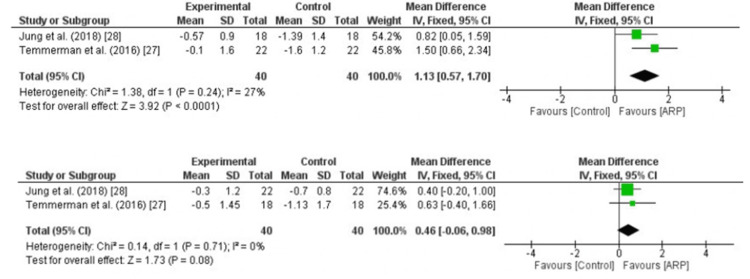
Forrest plot depicting pooled effect estimates of vertical dimensions measured at the buccal and palatal sides at three months Jung et al. (2018) [28]; Temmerman et al. (2016) [27]

Vertical dimensional changes: External changes were analyzed with an outcome of three months. The vertical height measured at the buccal and palatal sides at three months obtained by pooling two studies (Temmerman et al. (2016) [27] and Jung et al. (2018) [28]) was analyzed, and the effect size was 1.13 (0.57, 1.70) and 0.46 (-0.06, 0.98), respectively (Figure 4). There were 40 study participants, accounting for 80 extraction sockets.

Internal changes: Outcome of six months. The studies could technically not be pooled because there was actual weight gain in one study. The results were contrary to one another. The mean difference for the vertical height/socket depth six months postoperatively amounted to a gain of 3.37 (2.06, 4.68). This study (Madan et al. (2014) [30]) involved 30 patients, accounting for 60 extraction sockets. In the second study (Lekovic et al. (1998) [36]) with a sample size of 16 with 32 participants, the bone loss amounted to -1.87 (-2.09, -1.65).

Qualitative Analysis

Five studies with a total of 176 participants and 168 extraction sockets were used to do a qualitative analysis of the primary and secondary outcomes. The bone density, hard tissue dimensional changes, and histologic changes were analyzed.

Five studies were included. The bone dimensional changes in the studies conducted by Alsayed et al. (2020) [35] and Karaca et al. (2015) [32] were higher in the test than in the control at six months. The difference between the control and test groups at six months was statistically significant [35]. The remaining studies conducted at eight weeks, three months, and four months also favored socket preservation (Suttapreyasri et al. (2013) [29], Crespi et al. (2009) [31], and Araujo-Pires et al. (2016) [33]). The bone density at six months was comparably higher in the test than the control, attesting to better mineralization in the experimental group [35].

One study has shed some light on the histologic findings for the presence of vital bone, connective tissue, and residual graft material. The percentage of vital bone seemed to be higher, while the connective tissue and residual graft percentages were lower in the test than in the control, suggesting improved new bone formation in the preservation group [31].

Risk of Bias Within the Studies

**Figure 5:**
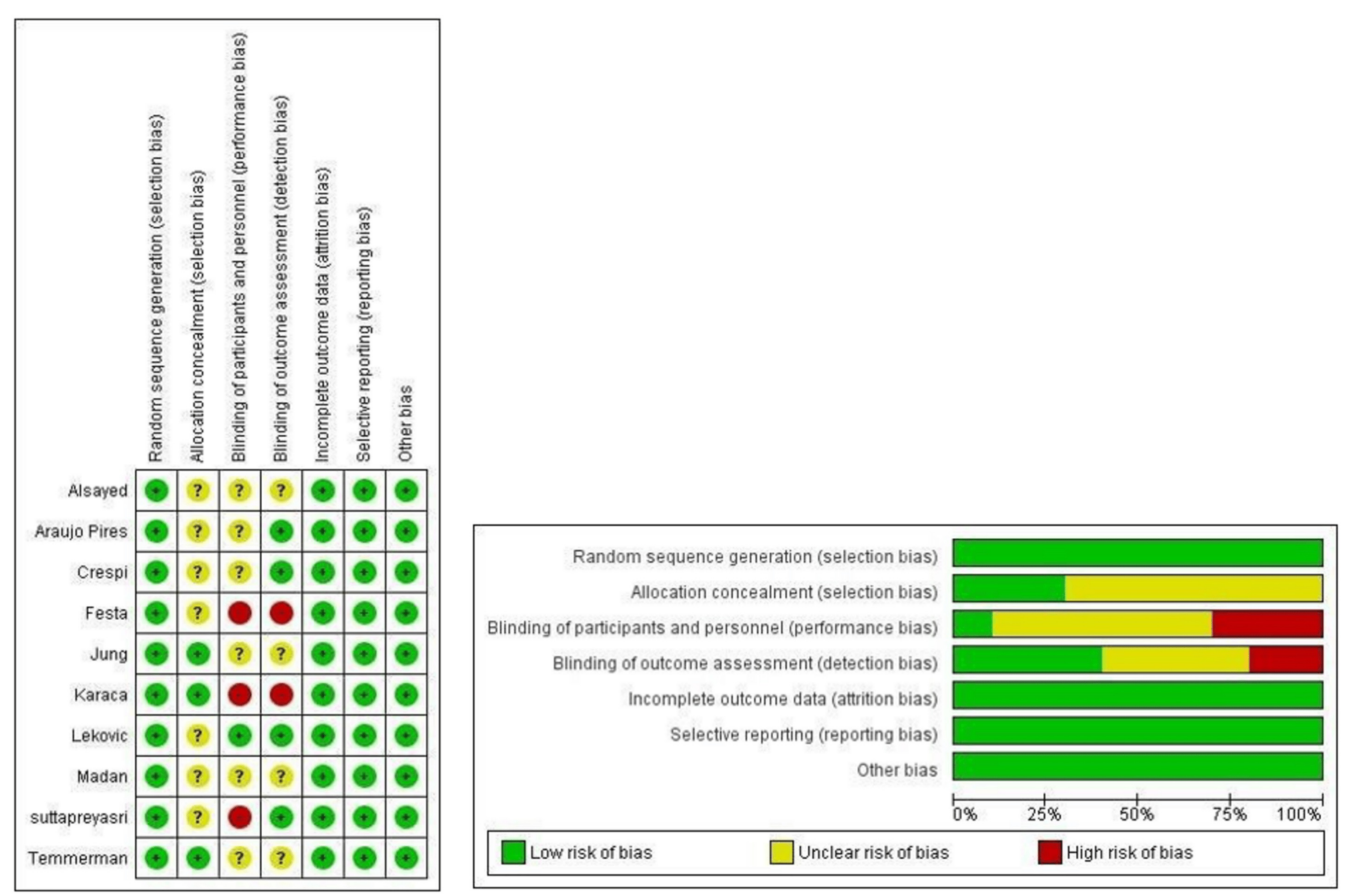
Summary of risk of bias assessment of included studies Alsayed et al. (2020) [35]; Araujo-Pires et al. (2016) [33]; Crespi et al. (2009) [31]; Festa et al. (2011) [34]; Jung et al. (2018) [28]; Karaca et al. (2015) [32]; Lekovic et al. (1998) [36]; Madan et al. (2014) [30]; Suttapreya sri et al. (2013) [29]; Temmerman et al. (2016) [27]

Low risk was present for all 10 studies concerning random sequence generation, allocation concealment, incomplete outcome data, and selective reporting. Computer-generated random sequence generation and the flip of a coin were the randomization methods employed. For allocation concealment, sealed opaque envelopes containing the list were opened strictly after the extraction and removal of the granulation tissue. Attrition bias was considerably low for the studies because of the general fact that differential attrition is eliminated as there could be no unequal dropouts in the split-mouth studies. The confounders were precisely the same for both the test and control groups. There was no evidence of overall attrition in the included studies except for one study in which the patients were withdrawn [28]. However, a sufficient number of patients were recruited, considering dropouts. The reasons for non-inclusion were elaborated, and the chances for it to cause significant bias were very low. The assessment of studies for bias due to selective reporting yielded fewer concerns. Other types of bias, such as baseline imbalance, recruitment bias, differential diagnostic activity bias, and bias due to external and internal validity, were less. However, since this is a surgical procedure, blinding of personnel is difficult, and in most of the published articles, it was not explained clearly. However, in one study, the surgeries for the test and control groups were performed by two surgeons, and the re-entry surgery was performed by two others [36]. The risk of bias assessment for the included studies is depicted in Figure 5.

Discussion

Bone remodeling after the loss of teeth is well documented. The resulting decrease in the hard and soft tissue volume differs among the population. This physiological process is affected by anatomic, biochemical, environmental, and biomechanical factors [37,38]. When considering implant replacement, the implant body selection is critical and depends on the available bone width and height. Ideally, the implant diameter and length should equal the root dimensions of the natural tooth it replaces. However, post-extraction alveolar bone resorption complicates treatment planning [5].

Bone healing and remodeling are significant only during the first year and become nil or minimal thereafter. Moreover, most of the bone loss occurs within the first three months. Some studies have reported actual bone gain after six months. In some reviews, the follow-up period entitled ranged between months and was pooled. In some systematic reviews, a period of less than six months was excluded. Since there is a sequential progression of events, in this study, the weighted mean difference for the first three months was calculated separately from that of the effect size after six months of alveolar preservation [39,40].

The results of the vertical ridge dimension were analyzed both externally and internally. The former is the buccal and palatal height, and the latter is the socket depth. The external ridge reduction for the vertical dimension was minimal in the group that received an intervention.

The results of this study, in terms of the horizontal dimension, showed a favorable shift toward ARP in comparison with the control. The results from the studies conducted by Avila Ortiz et al. (2019) [41], Troiano et al. (2018) [42], and Vignoletti et al. (2012) [43] have shown similar findings. The pooled effects in these studies were recorded over three to 12 months. Moreover, the width contraction was more pronounced at six months than at the three-month follow-up, indicating ongoing remodeling. In a review conducted by Tan et al. (2012) [40], the percentage difference was 29%-63% at six months, compared to 32% at three months.

The socket depth or the internal vertical measurement yielded two contrary results. Pooling these studies posed two difficulties, the first being the bone gain that occurred in the first study, which could easily lead to a statistical error. The first study conducted by Madan et al. (2014) [30] showed an actual bone gain. In the second study, there was a bone loss, and the weighted mean difference favored control by an effect estimate of -1.87 (-2.09, -1.65) [36]. A larger number of studies have to be analyzed to solve this controversy. Although the socket was grafted immediately, the level of grafting the socket was 1 mm above the socket, which is different from that of the conventional method and may also be the reason behind this problem. However, this could be due to any reason, as there is no conclusive evidence. Some studies have reported similar bone gain. In the study conducted by Schropp, he reported that bone gain at six months was more significant than the gains at three and twelve months [39]. The results from this study indicate that the reduction in width is more pronounced when compared to the reduction in height. Even in the vertical dimension, the pooled effect for changes on the buccal side shows more loss than bone loss on the palatal side [43]. The results from the qualitative analysis support the conclusions derived from the meta-analysis. The hard tissue dimensional changes in bone density and immunohistochemistry studies do not show any notable deviations. All the included studies were split-mouth; hence, the inter-subject variability was considerably reduced. The split-mouth randomized control trial design was adopted to minimize statistical heterogeneity. The symmetrical arrangement of teeth in relation to the sagittal plane along with equal distribution in all four quadrants strategically spread along different planes, allows split-mouth trial designs in dentistry. This design is superior to parallel arm trials in that it reduces inter-subject variability in terms of gender, local factors, and systemic factors [44]. However, there are also drawbacks. Hence, it has to be dealt with caution to account/confound for these. Some negative aspects, such as external validity and internal validity, were dealt with while choosing eligible articles [45]. The articles included in this review did not stick to a split-mouth mirror model; a multi-level model including all quadrants of the mouth, three-arm designs, and different randomizations could be seen in the selected articles, thereby reducing bias. Any carry-over effect via leakage was not possible in this type of intervention.

Limitations

The number of studies included in the meta-analysis was less. Quite a few studies that could have yielded valuable results were rejected because of the placement of a membrane in the control group. Moreover, a meta-regression for three months, four months, six months, and twelve months could provide insight into the events occurring at specific periods. A funnel plot could not be performed owing to the lower number of studies. The quality of some studies ranged from low to moderate. It was possible for selective exclusion due to the design of the study. Patient correlation data were not provided for the included studies. The lack of randomized controlled trials in terms of additional outcomes was an issue, as it could have broadened the horizons. Several clinical variations, such as the presence of adjacent teeth, gingival biotype, variation in surgical techniques, and the presence or absence of sutures, could have influenced the results.

## Conclusions

Within the limitations of this study, it can be safely concluded that ARP minimizes bone loss. The horizontal bone loss at six months is much more than the reduction at three months, suggesting an ongoing or active phase of remodeling. Regarding the height, the external vertical dimension shows preservation compared to unassisted socket healing. In the future, more homogenous studies must be undertaken and pooled for a better understanding of the changes in the socket depth. Hence, there is a need for a review with all possible outcomes combined while minimizing clinical and statistical heterogeneity.
